# Transient middle cerebral artery occlusion with complete reperfusion in spontaneously hypertensive rats

**DOI:** 10.1016/j.mex.2014.11.001

**Published:** 2014-11-11

**Authors:** Jeff W. Hill, Edwin M. Nemoto

**Affiliations:** University of New Mexico Health Sciences Center, Department of Neurosurgery, Albuquerque, NM 87131, United States

**Keywords:** Transient middle cerebral artery occlusion with complete reperfusion, Middle cerebral artery occlusion, MCAO, Stroke, Reperfusion, Spontaneously hypertensive rat, SHR

## Abstract

Middle cerebral artery occlusion (MCAO) by the intraluminal suture method is widely used to model ischemic stroke in rats. Current methods include transection or ligation of the external carotid or common carotid artery and thus result in partial restoration of perfusion after transient MCAO. Since incomplete reperfusion may influence recovery and thus confound studies of the impact of neuroprotective compounds and therapies on outcomes after stroke, we have devised a novel method to induce transient MCAO with complete reperfusion. Advantages of the method include:

• MCAO is achieved through insertion of an intraluminal suture into the internal carotid artery through the common carotid artery.
• At the end of the occlusion period, the suture is withdrawn and the incision in the common carotid artery is closed with cyanoacrylate tissue adhesive and complete reperfusion is established.
• No residual subcutaneous sutures remain during recovery.
• Vasculature is restored to the preoperative state.

## Method

### Required materials

Spontaneously hypertensive rats (SHR) (male, 290–300 g, Harlan or Charles River Laboratories)

MCAO sutures (Doccol Corporation, 403923PK10)

4–0 braided silk sutures (Ethicon, A183H)

S&T vascular clamp, 7 mm length, 1 × 3.5 mm jaws (Fine Science Tools, 396-01)

Colibri Retractor, 1.5 cm spread, 9 × 3 × 6 cm teeth (Fine Science Tools, 17000-03)

GLUture topical tissue adhesive (32406-01)

## Method details

All animal procedures were approved by the University of New Mexico Institutional Animal Care and Use Committee and were in compliance with federal guidelines. Animals were anesthetized with 2% isoflurane inhalant during all surgical procedures.

### Step one: preparation of the common carotid artery (CCA) for insertion of the occluding suture

The animal is anesthetized with 2% isoflurane and immobilized in the supine position on a surgical table with a 15 ml tube placed under the neck to facilitate visualization of neck vasculature. The neck is shaved, disinfected with povidone-iodine solution, and cleaned with 70% ethyl alcohol. A shallow 25 mm incision is made along the midline from the base of the mandible to the sternum. The right CCA is carefully exposed while avoiding injury to soft tissues and nerves as described elsewhere [1]. The sternohyoid and sternomastoid muscles are retracted and the CCA is separated from the vagus nerve (Fig. 1). The CCA is ligated with a 4–0 silk suture with a single knot 6–7 mm caudal to the bifurcation of the CCA into the external carotid artery (ECA) and internal carotid artery (ICA). The knot should be sufficiently tight to occlude the CCA but not overly tightened so as to prevent injury to the CCA and facilitate later removal of the suture. A vascular clamp is placed on the CCA immediately caudal to the bifurcation. Two 4–0 silk sutures are tied loosely around the CCA between the first suture and the vascular clamp (Fig. 2).

### Step two: middle cerebral artery occlusion (MCAO)

A punctate incision is made in the ventral wall of the CCA with a 25G needle 5 mm caudal to the clamp and a 4–0 nylon suture (Doccol Corporation) 30 mm in length with a 2–3 mm silicone-coated tip (0.39 mm diameter) is advanced into the CCA lumen to the clamp. The lower silk suture is secured around the occluding suture to prevent bleeding (Fig. 3). The clamp is removed and the suture advanced into the ICA until resistance is felt. In the correct position, the end of the suture should be roughly at the caudal end of the surgical field. If resistance is felt while much of the suture is outside the surgical field, the suture may have entered the pterygopalatine artery. If this occurs, the suture is pulled back to the bifurcation and reinserted while attempting to direct it toward the midline. To ensure proper placement of the occluding suture once resistance is felt, the suture is grasped with forceps, bent toward the midline, and gently pushed forward into the ICA until flexion of the suture can be visualized through the lateral wall of the ICA (Fig. 4). Care must be taken during this step to not insert the suture pass the point of visualizing flexion through the ICA or the ICA may be punctured by the suture and result in subarachnoid hemorrhage. Additionally, flexion of the occluding suture should be visualized only once to prevent stretching of the ICA which can result in increased variability in infarction. After flexion of the suture has been observed, the suture is immediately secured in place by tightening the two silk sutures around the CCA and trimming off the end of the occluding suture with micro scissors (Fig. 5). In this position, the suture occludes the middle cerebral artery (MCA) origin and MCA territory becomes ischemic. Note the position of the end of the occluding suture in relation to the caudal suture occluding the CCA. This observation is useful in determining if any movement of the occluding suture occurred during the occlusion period. The wound is closed and the animal is allowed to recover during the 90-min occlusion period.

### Step three: removal of the occluding suture and complete restoration of perfusion

After 90 min, the animal is reanesthetized and the surgical field exposed. The upper suture is loosened and the occluding suture is withdrawn to the middle suture. The upper suture is moved rostrally and secured around the CCA above the silicone tip of the occluding suture. The middle and occluding sutures are removed and the CCA is clamped 1 mm rostral to the incision in the CCA (Fig. 6). In this position, the incision is visible on the surface of the CCA and the CCA lumen is closed. A small drop of tissue adhesive is applied to the back of a 25G needle by placing the tip of the needle bevel up in a drop of adhesive. The proper amount of adhesive (an approximately 0.25 mm thick drop) can be verified through the microscope before application to the incision (Fig. 7). The adhesive is applied directly onto the incision (Fig. 8) and the clamp is removed after 1 min. Using a clean 25G needle, a second drop of adhesive is applied around the perimeter of the first application to create a patch approximately 0.5 mm in diameter (Fig. 9). The adhesive is allowed to cure for 2 min. The rostral silk suture around the CCA is loosened and the patch is observed for leakage. If leakage around the patch is detected, the rostral silk suture is resecured and the patch is expanded in the affected area with a minimal application of adhesive. If the patch is secure, the lower suture is carefully removed and perfusion through the CCA is verified (Fig. 10). After complete reperfusion is achieved (Fig. 11), the wound is closed and the animal is given 3 ml of subcutaneous normal saline to prevent dehydration.

## Additional information

### Representative outcome after transient MCAO with complete reperfusion

Twenty-four hours and 7 days after the complete restoration of perfusion after a 90-min MCAO, infarction and edema were examined by T2-weighted magnetic resonance imaging (MRI). After 24 h reperfusion, standard deviation in mean infarct volume in a group of 8 animals undergoing MCAO with complete perfusion was less than 10% (Fig. 12). Significant cortical and striatal infarction and edema typical of intraluminal suture-induced MCAO were observed (Fig. 13A). After 7 days of complete reperfusion, significant recovery, as indicated by visible reductions in edema and infarction, was observed (Fig. 13B). After MRI at 7 days post-complete reperfusion, the CCA was exposed to examine the patch site. The patch was intact and integrated with the CCA with no indications of inflammation, fibrosis, or foreign body reaction (Fig. 14). Careful removal of the patch with forceps revealed a fully intact and regenerated CCA (Fig. 15).

### Background

Transient occlusion of the middle cerebral artery by the insertion of an intraluminal suture is a widely used and accepted animal model of ischemic stroke. In the method described by Longa et al., the ECA is permanently transected and the occluding suture is inserted into the ICA via the ECA stump to occlude the origin of the MCA [2]. The method represented a significant advancement in ease of implementation compared to occluding the MCA origin by craniotomy. Studies have since shown that loss of perfusion through the ECA causes lesions in masticatory muscles [3]. Accordingly, transection of the ECA may influence feeding behavior after MCAO and potentially confound studies of recovery and neurological outcome after stroke. In the ECA-entry method, the superior thyroid (ST) and occipital arteries (OA) are typically permanently ligated. Permanent loss of perfusion through these vessels may also influence recovery and responses to neuroprotectants.

CCA-entry intraluminal suture MCAO was shown to provide more consistent infarction than ECA-entry MCAO, presumably through the increased ease of suture insertion through the CCA and added ability to feel proper insertion of the occluding suture due to alignment of the suture with the ICA [4]. Previously reported CCA-entry intraluminal suture MCAO studies have utilized permanent ligation of the ipsilateral CCA to close the occluding suture entry-point incision [5]. In this model, reperfusion of the ischemic hemisphere is achieved through the circle of Willis. While the method provides infarction without ligation of the ECA, ST, and OA, the extent of reperfusion may be variable among animals with decreased functionality of collateral circulation, such as in the widely used SHR and SHRSP (stroke-prone spontaneously hypertensive rat) strains. Further, permanent CCA ligation may result in embolization of thrombotic material present in the CCA on either side of the ligation [6].

A limitation of both methods is that subcutaneous silk sutures used to permanently occlude vessels remain in the animal throughout recovery. While silk is a suture material of choice for neurological procedures due to its low cost, pliability, tensile strength, and long-term stability, it is proteinaceous and elicits a significant and rapid acute inflammatory response. Subcutaneous silk sutures typically become inflamed with hours to days of implantation and gradually become encapsulated with fibrous connective tissue. Since neurological injury during stroke is an inflammatory process, the immunological reaction to silk sutures remaining on the vasculature in intraluminal suture MCAO techniques may contribute to variability in infarction and confound the response to neuroprotective compounds, many of which possess anti-inflammatory properties.

A previous study reported limited success in closing microvascular lesions in rats using fibrin sealant [7]. Fibrin sealant, or fibrin glue, is a mixture of fibrinogen, thrombin, and clotting factors which initiates the formation of a fibrin clot at the application site. A substrate, such as a muscle pad, is required and the clotting agents are prone to enter the vessel lumen and cause intravascular thrombosis. The study reported significant rates of stenosis and intraluminal thrombosis in the closure of CCA incisions with fibrin sealant. Surgical outcomes with the use of fibrin sealant for CCA closure may be further confounded by the inclusion of a muscle pad or other substrate which may precipitate a foreign body reaction. Additionally, fibrin sealants do not provide the immediate sealing and superior tensile strength of cyanoacrylate tissue adhesives and thus are less well suited for closure of highly pressurized vessels such as the CCA in time-sensitive MCAO procedures.

With the limitations of current MCAO and microvascular closure methods, we sought to achieve MCAO with complete reperfusion without the use of fibrin sealant or the presence of residual subcutaneous silk sutures during recovery. We demonstrate that the use of cyanoacrylate tissue adhesive is an effective method for closing the CCA after removal of an occluding suture. Cyanoacrylate tissue adhesive has been shown to elicit significantly less inflammation than silk sutures in rats [8]. No site reactions have been observed in animals undergoing MCAO with complete reperfusion as described in this manuscript and consistent and significant infarction and recovery have been observed. Since infarction is inversely correlated with neurological outcome and perfusion of mastication muscles is unaffected in the refined method, we anticipate that the method may reduce inter-animal variations in neurological outcome measures related to infarction and feeding behavior. MCAO with complete reperfusion offers several advantages over previously reported methods and may represent a significant refinement to existing animal stroke models.

## Figures and Tables

**Fig. 1:**
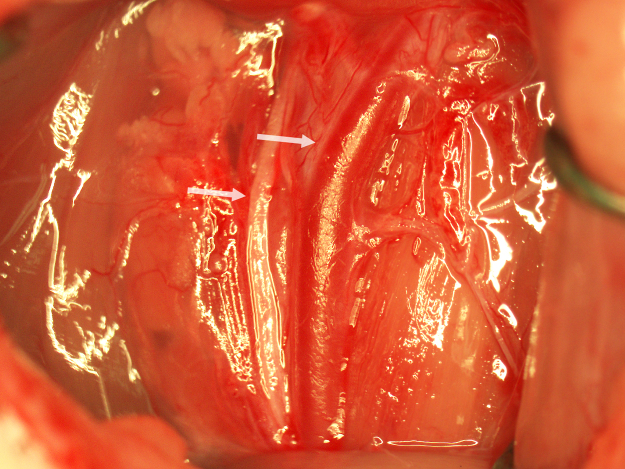
The retracted surgical field showing the bifurcation of the CCA (upper arrow) and the vagus nerve (lower arrow).

**Fig. 2:**
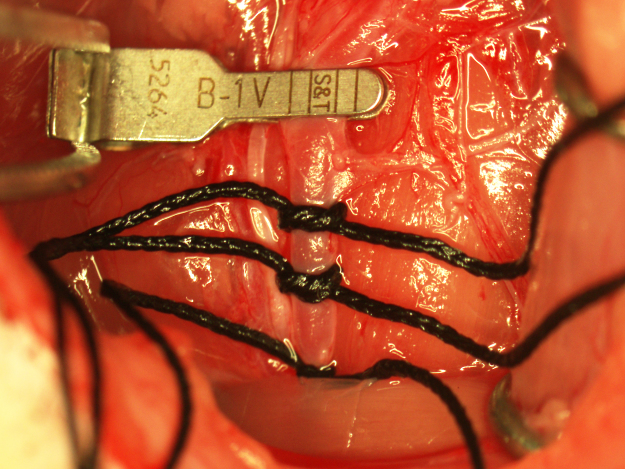
Preparation of the CCA for occluding suture insertion.

**Fig. 3:**
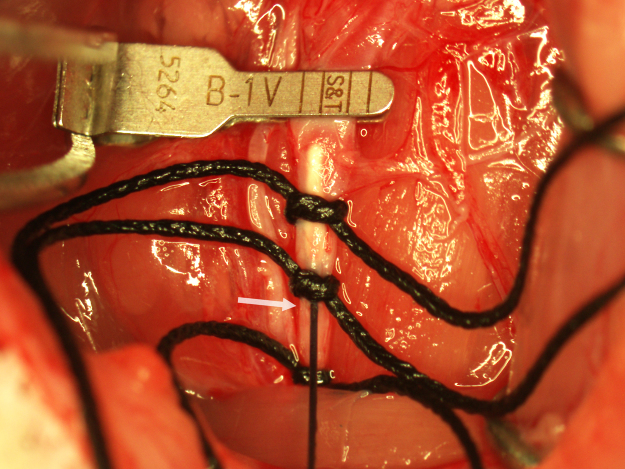
Insertion of the occluding suture. The punctate incision in the CCA is indicated with an arrow.

**Fig. 4:**
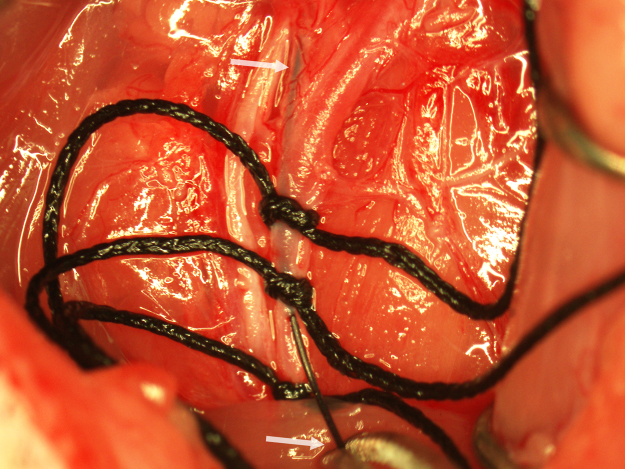
Verification of proper insertion of the occluding suture. The suture is held on the end with forceps (lower arrow) and bent toward the midline while gently advancing it into the ICA until flexion of the suture is observed through the lateral wall of the ICA (upper arrow).

**Fig. 5:**
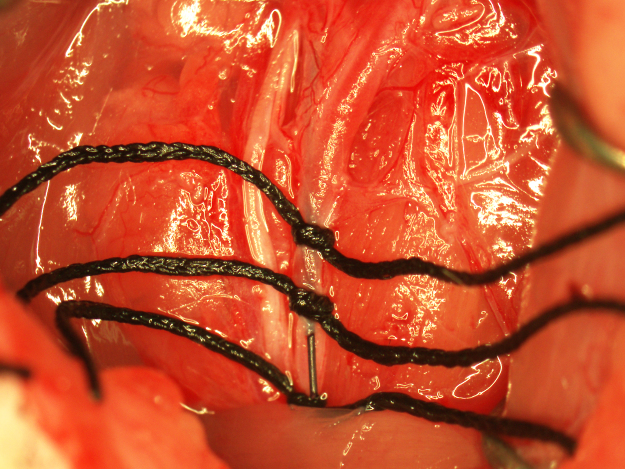
MCAO with a properly secured occluding suture.

**Fig. 6:**
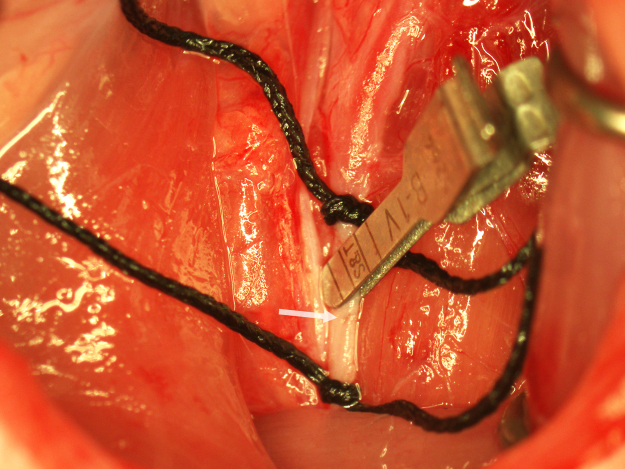
Preparation of the CCA for closure with cyanoacrylate adhesive. The CCA is clamped to expose the punctate incision in the ventral wall of the CCA (arrow) and prevent entry of adhesive into the CCA lumen.

**Fig. 7:**
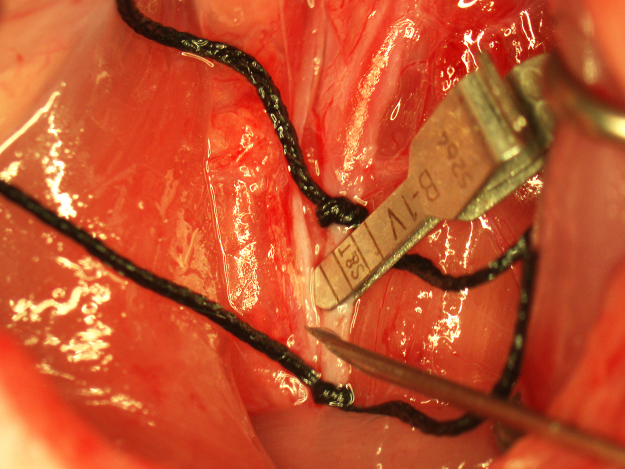
Application of tissue adhesive to the CCA. Adhesive is applied to the CCA incision using a 25G needle.

**Fig. 8:**
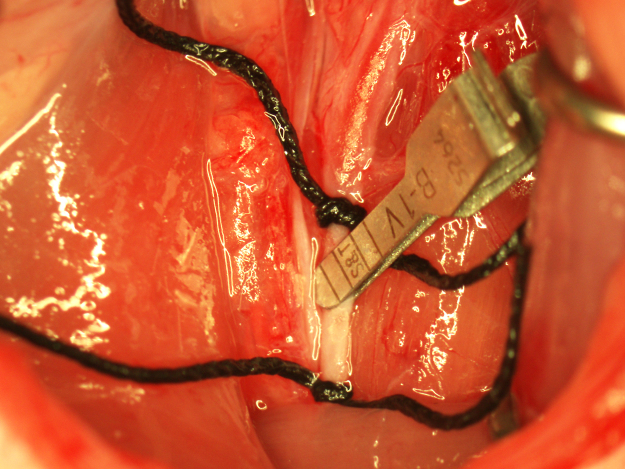
Closure of the CCA incision after the first application of tissue adhesive.

**Fig. 9:**
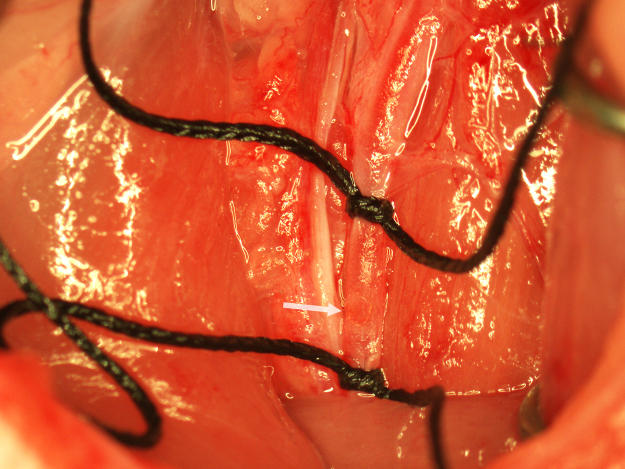
Complete closure of the CCA after a second application of adhesive. The cyanoacrylate patch is indicated with an arrow.

**Fig. 10:**
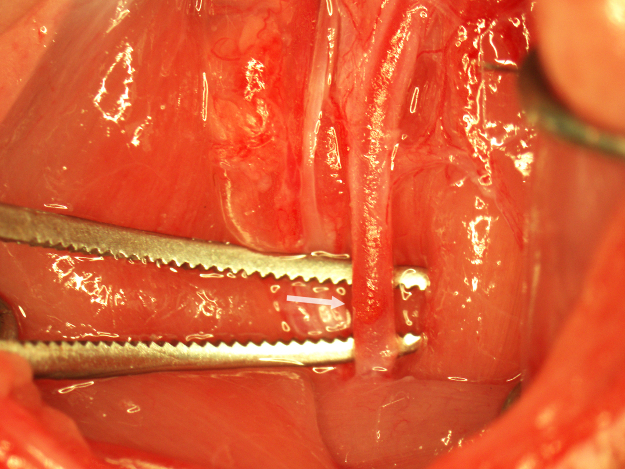
Verification of perfusion through the CCA. The patched CCA (indicated with an arrow) is lifted with forceps and observed for perfusion.

**Fig. 11:**
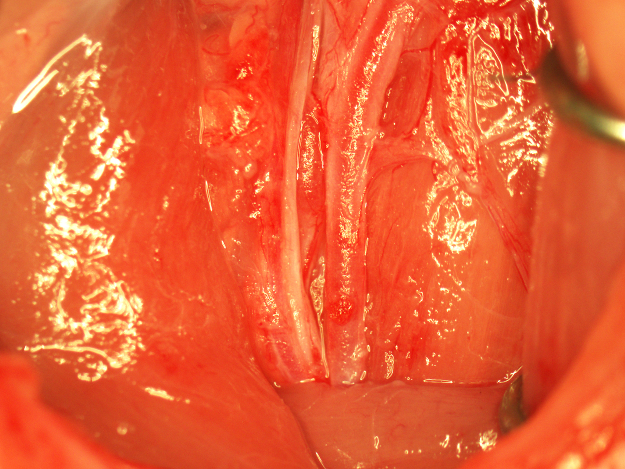
Completion of MCAO with complete reperfusion.

**Fig. 12:**
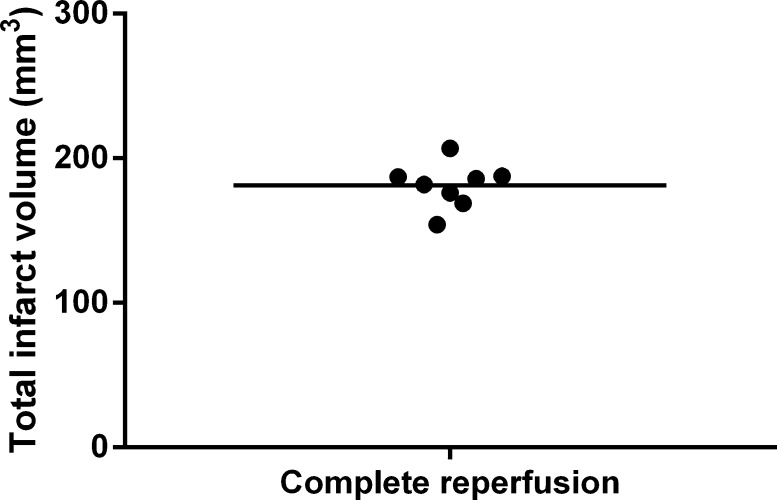
Infarct volume after a 90-min MCAO and 24 h complete reperfusion. Mean infarct volume was 181.0 ± 15.5 mm^3^ (*n* = 8 animals).

**Fig. 13:**
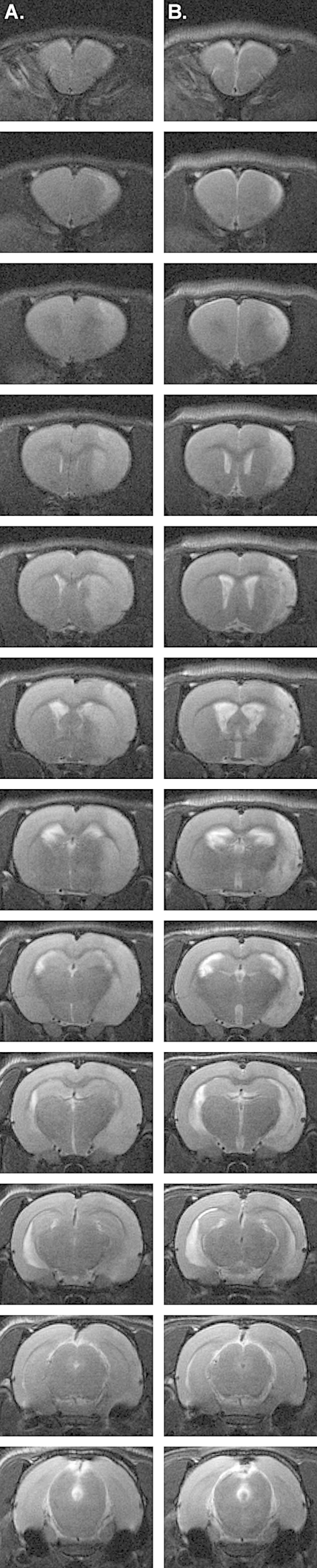
T2-weighted MRI performed 24 h (A) and 7 days (B) after MCAO with complete reperfusion.

**Fig. 14:**
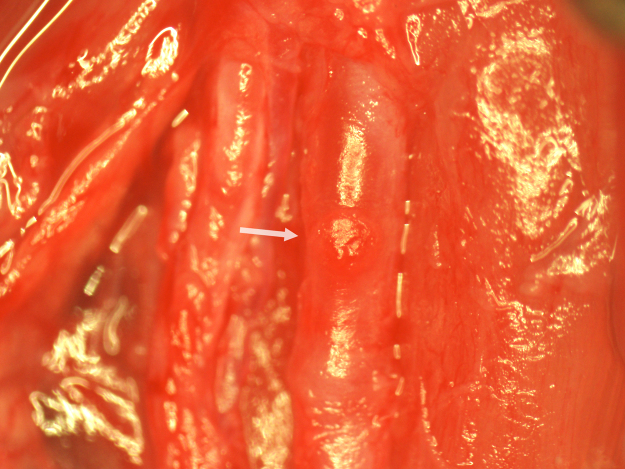
Examination of the CCA patch site after 7 days of recovery.

**Fig. 15:**
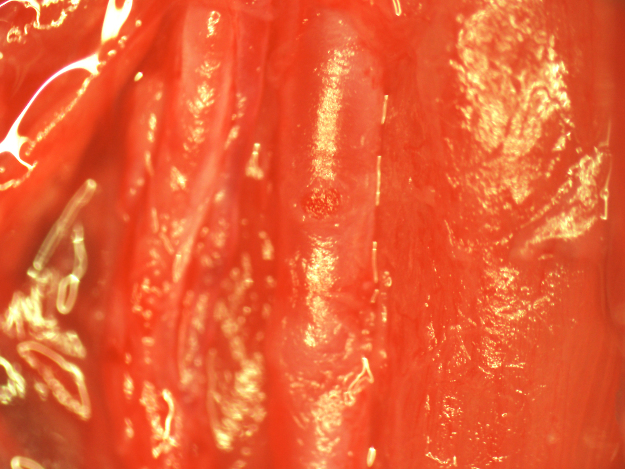
Removal of the CCA patch after 7 days of recovery.
